# Supplementary material for: Chromothripsis during telomere crisis is independent of NHEJ, and consistent with a replicative origin
Source: Genome Res. 2019 May;29(5):737–49. doi: 10.1101/gr.240705.118 (PMC6499312; doi:10.1101/gr.240705.118)
Supplement: Supplemental Material [file supp_gr.240705.118_Supplemental_file_1.zip › contigs/annotated_contigs/DB79/contig.2.DB79_length_286_mean_cov_2.62237762238.docx]

**DB79_length_286_mean_cov_2.62237762238**

```
CATTCAACGAACATTTAATGAATGCCTACTATGTGTCAGACAGG|TA|TCAAGAGAAGCAAAACAACACATGGTAGGTTCAGAGGGAAA
 >chr7:129431724-129431770 - E=2e-16         >chr7:7081329-7081571 + E=7e-135
GCTGAGGGCAGCATCTCTGTAATTTTTGAGATTCGTGGATAGCCTCTCTAATTACGAATATCTCAAAATTGTGCAAAACAGAGAATACA

TTTGCTGGCAAGATGGCCGAATAGGAAGAGCTCTGGTCTGCAGCTCCCAGTGAGATCGACACAGAAGGCAGGTGATTTCTGCATTTCCA

ATTGAGGTACCCGGTTCATCT
```
